# Single-cell multiomics analyses of spindle-transferred human embryos suggest a mostly normal embryonic development

**DOI:** 10.1371/journal.pbio.3001741

**Published:** 2022-08-16

**Authors:** Shuyue Qi, Wei Wang, Xiaohui Xue, Zhuo Lu, Jia Yan, Yunfei Li, Yu Zhang, Mingming Shu, Chunlan Song, Qihang Wang, Yunhai Chuai, Xinyu Zhai, Shujie Han, Fuchou Tang, Wei Shang

**Affiliations:** 1 Biomedical Pioneering Innovation Center, School of Life Sciences, Peking University, Beijing, China; 2 Department of Obstetrics and Gynecology, The seventh Medical Center of Chinese PLA General Hospital, Beijing, China; 3 Department of Obstetrics and Gynecology, Chinese PLA General Hospital, Beijing, China; 4 Peking University-Tsinghua University-National Institute of Biological Sciences Joint Graduate Program, Academy for Advanced Interdisciplinary Studies, Peking University, Beijing, China; 5 Beijing Advanced Innovation Center for Genomics, Ministry of Education Key Laboratory of Cell Proliferation and Differentiation, Beijing, China; 6 New Hope Fertility Center, New York, New York, United States of America; 7 Peking-Tsinghua Center for Life Sciences, Academy for Advanced Interdisciplinary Studies, Peking University, Beijing, China; 8 Department of Histology and Embryology, Hebei Medical University, Shijiazhuang, Hebei, China; 9 Department of Obstetrics and Gynecology, The sixth Medical Center of Chinese PLA General Hospital, Beijing, China; 10 Navy Clinical Medical School, Anhui Medical University, Hefei, Anhui, China; 11 South China University of Technology, Guangzhou, Guangdong, China; Academia Sinica, TAIWAN

## Abstract

Mitochondrial DNA (mtDNA) mutations are often associated with incurable diseases and lead to detectable pathogenic variants in 1 out of 200 babies. Uncoupling of the inheritance of mtDNA and the nuclear genome by spindle transfer (ST) can potentially prevent the transmission of mtDNA mutations from mother to offspring. However, no well-established studies have critically assessed the safety of this technique. Here, using single-cell triple omics sequencing method, we systematically analyzed the genome (copy number variation), DNA methylome, and transcriptome of ST and control blastocysts. The results showed that, compared to that in control embryos, the percentage of aneuploid cells in ST embryos did not significantly change. The epiblast, primitive endoderm, and trophectoderm (TE) of ST blastocysts presented RNA expression profiles that were comparable to those of control blastocysts. However, the DNA demethylation process in TE cells of ST blastocysts was slightly slower than that in the control blastocysts. Collectively, our results suggest that ST seems generally safe for embryonic development, with a relatively minor delay in the DNA demethylation process at the blastocyst stage.

## Abstract 2

Uncoupling of the inheritance of mtDNA and the nuclear genome by spindle transfer could prevent the transmission of mtDNA mutations. Systematic single-cell multiomic analyses of spindle transferred human embryos suggest this technique seems generally safe for human embryonic development and deserves further scientific evaluation and clinical testing.

## Introduction

Mitochondria are indispensable organelles of most eukaryotic cells and play an essential role in numerous metabolic processes such as oxidative phosphorylation, apoptosis, and calcium homeostasis. Mitochondria are also the only organelles in humans with their own genetic material, which includes 37 genes with 13 encoding mitochondrial proteins and 24 generating noncoding RNAs. Mutations in mitochondrial DNA (mtDNA) may cause mitochondrial dysfunction and lead to debilitating or devastating diseases that can affect virtually any organ at any age [1,2]. Mitochondrial diseases are among the most common inherited metabolic diseases [3,4], with 1 in 5,000 being the minimum prevalence rate for mtDNA mutations in adults [5]. Moreover, mitochondrial genome is invariably maternal inheritance [6], and at least 1 in 200 offspring of female carriers carry an mtDNA mutation that potentially causes disease [7]. Prenatal and preimplantation genetic diagnosis are available reproductive options for preventing the intergenerational transmission of mtDNA mutation-related diseases. However, these alternatives are only appropriate for women who can produce oocytes and embryos with low enough percentage of mutant mtDNAs [8].

To prevent the transmission of mitochondrial disease through the germline, clinicians have developed several different strategies for mitochondrial replacement therapy, including pronuclear transfer [9,10], spindle transfer (ST) [11,12], and polar body transfer [13–15]. The core principle of the abovementioned methods involves uncoupling the inheritance of mtDNA from that of the nuclear genome through the combination of nuclear DNA from oocytes or zygotes of a woman with mutated mtDNA with the cytoplasm of oocytes or zygotes from a healthy donor to obtain reconstructed embryos with both a normal nuclear genome and normal mtDNAs; these techniques have been developed over the past few decades. Currently, mitochondrial replacement therapy, a type of germline gene therapy, seems to be the only promising way to block the inheritance of mtDNA mutation-related diseases [16,17].

Additionally, replacement of oocyte mutant mtDNA and normal development of offspring by ST was shown to be effective in nonhuman primates [12,18,19]. The results from several groups using human oocytes suggest that the development to the blastocyst stage and derivation of embryonic stem cells (ESCs) of ST embryos are indistinguishable from those of control embryos in vitro, with negligible mtDNA carryover (<1%) [18,20]. In addition, mitochondrial respiratory chain enzyme activities and oxygen consumption rates of isolated ESCs and differentiated cells of ST embryos were shown to be comparable to those of control [20–22]. These results suggest that isolating maternal metaphase II spindles for nuclear transfer can be accomplished with minimal mtDNA carryover and has potential clinical applications [23]. In April 2016, Zhang and colleagues used the ST procedure to deliver a live boy in Mexico whose mother’s mitochondria carried the mutation causing Leigh syndrome [24]. The boy is still under long-term observation, but to date, no systematic omics study has been implemented on the safety of the ST procedure in humans.

Recent studies have shown that single-cell sequencing has great potential and advantages for analyzing embryo characteristics [25,26]. In the present study, we report a single-cell triple omics sequencing analysis of the genome (copy number variation (CNV)), transcriptome, and DNA methylome of ST and control blastocysts.

## Results

**Fig 1:**
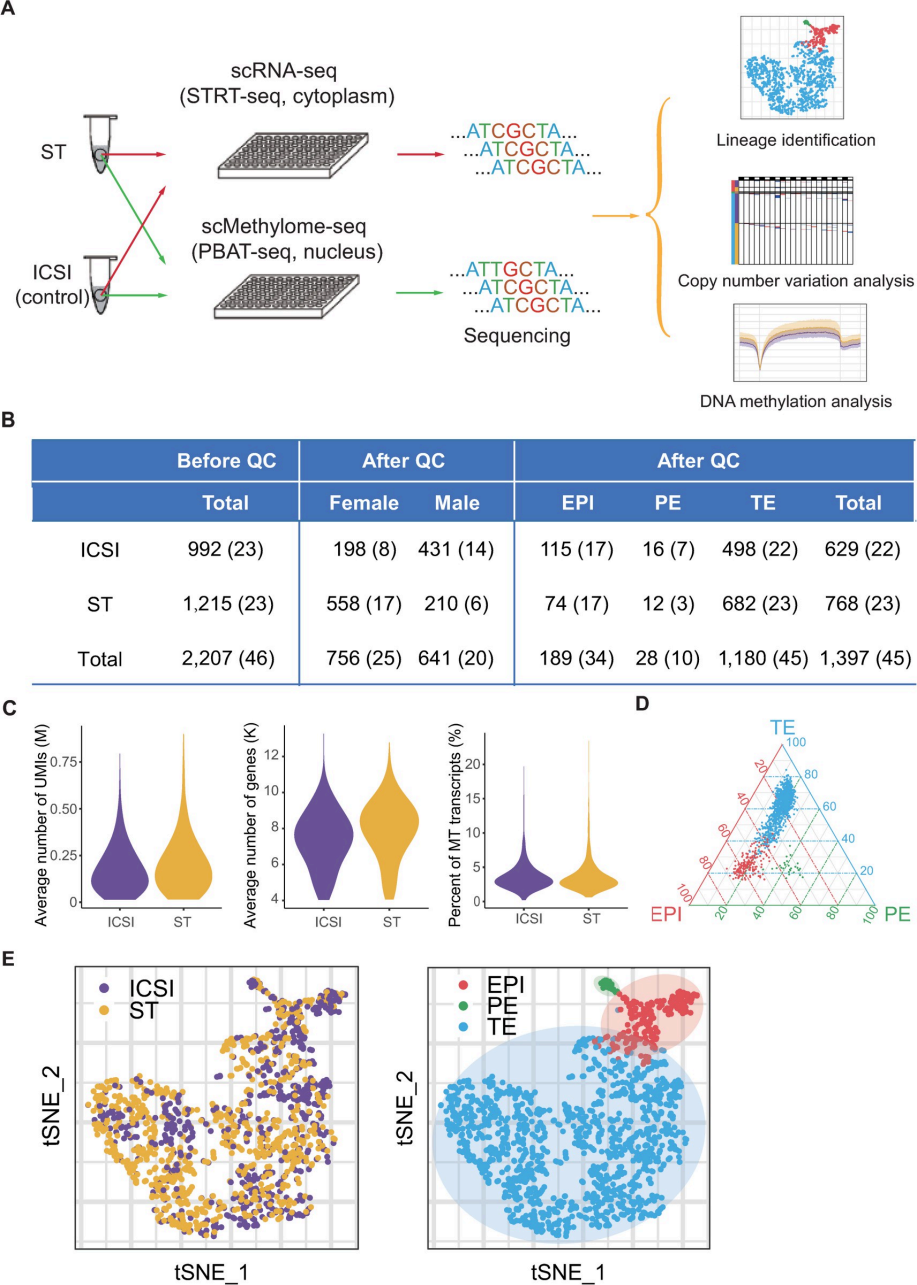
Single-cell Trio-seq2 sequencing of spindle-transferred human embryos. (A) Schematic of single cell collection, transcriptome sequencing, and DNA methylome sequencing and analysis. (B) Summary of the number of cells and embryos before and after quality control for the ICSI and ST groups. (C) Average number of UMIs and genes and percentage of MT transcripts in 1,397 cells passing quality control. (D) Ternary plots of lineage scores. (E) A total of 1,397 cells retained after quality control projected onto a t-SNE map and colored according to group (left) and lineage (right). The numerical data are listed in S1 Data. EPI, epiblast; ICSI, intracytoplasmic sperm injection; MT, mitochondrial; PBAT-seq, post-bisulfite adaptor tagging sequencing; PE, primitive endoderm; QC, quality control; scMethylome-seq, single-cell DNA methylome sequencing; scRNA-seq, single-cell RNA-seq; ST, spindle transfer; STRT-seq, single-cell tagged reverse transcription sequencing; TE, trophectoderm; t-SNE, t-distributed stochastic neighbor embedding; UMI, unique molecular identifier.

First, we collected 2,207 individual cells from 46 blastocysts (S1 Table) from the 2 experimental groups. After stringent filtration, 1,397 cells (768 cells from 23 ST blastocysts and 629 cells from 22 intracytoplasmic sperm injection (ICSI) control blastocysts) were retained for subsequent analyses (Fig 1A and 1B). On average, in each individual cell, we detected 7,683 expressed genes and 190,494 copies of mRNAs (Fig 1C).

**Fig 2:**
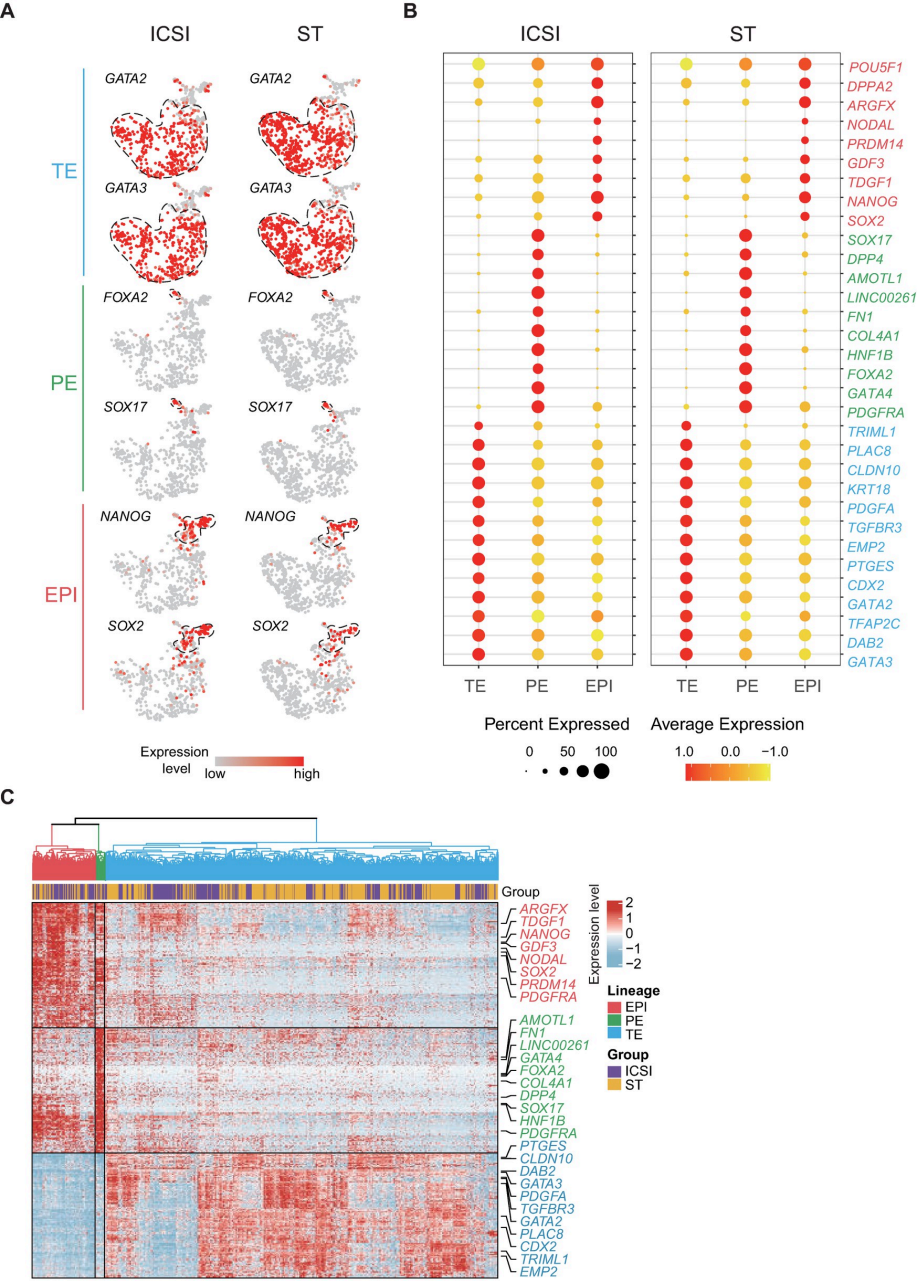
Lineage identification of scRNA-seq data. (A, B) The lineage identification results were verified by (A) the expression levels of high-confidence lineage markers and (B) the expression levels and percentages of lineage marker sets. (C) Expression patterns of lineage signature genes. Hierarchical clustering of 1,397 cells with Euclidean distance according to the Ward.D2 method. And lineage-specific genes were identified and intersected with known markers of the corresponding lineages. The numerical data are listed in S1 Data. EPI, epiblast; ICSI, intracytoplasmic sperm injection; PE, primitive endoderm; ST, spindle transfer; TE, trophectoderm.

Unsupervised t-distributed stochastic neighbor embedding (t-SNE) analysis revealed that these cells could be partitioned into 3 main clusters. According to the canonical markers and lineage scores, these clusters were determined to be epiblast (EPI), primitive endoderm (PE), and trophectoderm (TE). Lineage-specific marker genes were verified in our data, including *SOX2*, *NANOG*, and *POU5F1* (also known as *OCT4*) for EPI; *SOX17* and *FOXA2* for PE; and *GATA2*, *GATA3*, and *CDX2* for TE (Figs 1D and 1E and 2). For all 3 lineages, the cells from ST blastocysts and from ICSI blastocysts were tightly clustered in the t-SNE plot, indicating that the differentiation of these 3 lineages is generally normal in ST embryos (Fig 1E). Hierarchical clustering analysis based on the expression of lineage-specific genes also showed that the developmental potential of ST blastocysts was comparable for all 3 lineages of cells (Fig 2C).

**Fig 3:**
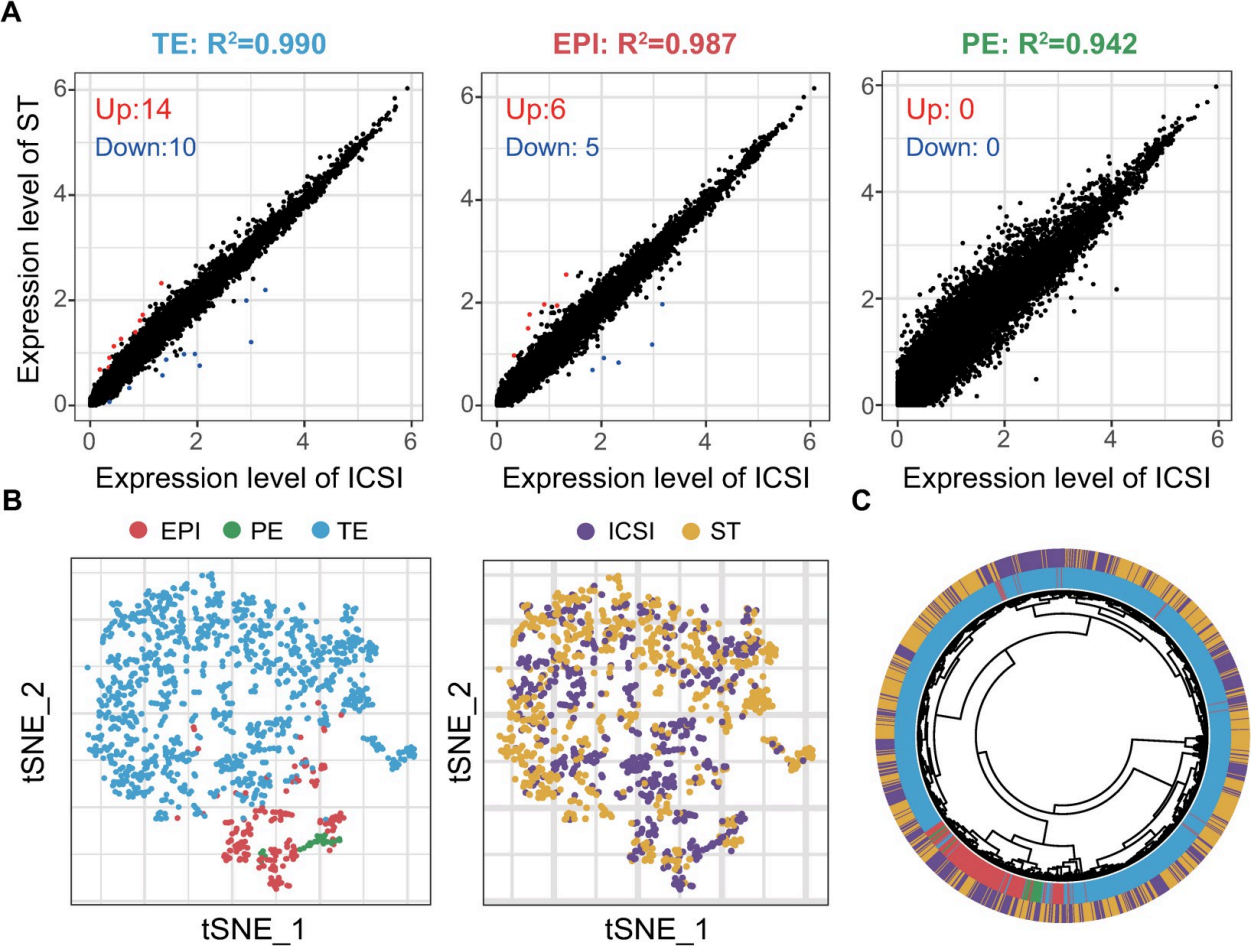
Comparative analysis of ST and ICSI control embryos. (A) Analysis of DEGs and linear regression analysis of ST and ICSI embryos. The ST-to-ICSI ratio of the mean expression level (ln(TPM/10+1)) for each commonly expressed gene was fitted to y~x. The correlation coefficient of each fitting and the number of up-regulated (red) or down-regulated (blue) DEGs are listed for each lineage. (B) ST and ICSI embryonic cells projected onto a t-SNE map of AUC scores from SCENIC analysis and colored according to lineage (left) and group (right). (C) Hierarchical clustering of AUC scores from SCENIC analysis. Cells of the same lineage (red, EPI; green, PE; blue, TE) clustered together. EPI and PE cells belonged to the same branch and converged to the root with TE branches. ST (yellow) and ICSI (purple) clustered together regardless of cell lineage. The numerical data are listed in S2 Data. DEG, differentially expressed gene; EPI, epiblast; ICSI, intracytoplasmic sperm injection; PE, primitive endoderm; SCENIC, single-cell regulatory network inference and clustering; ST, spindle transfer; TE, trophectoderm; t-SNE, t-distributed stochastic neighbor embedding.

Next, to explore the differences between the ST and control blastocysts in more detail, we analyzed the differentially expressed genes (DEGs). The patterns of gene expression were nearly identical for all of the TE, EPI, and PE lineages between the ST and control embryos. There were 24, 11, and 0 DEGs in the TE, EPI, and PE lineages, respectively (Figs 3A and S1A and S2 Table). Regression analysis showed that the ST and control blastocysts were highly similar between corresponding lineages, with high correlation coefficients (R^2^ values) for TE (0.990), EPI (0.987), and PE (0.942) (Fig 3A). Calculated variance (SD) of expression level for EPI, PE, and TE lineages in the ST and control ICSI embryos was similar in all 3 lineages and even lower in PE (S1C Fig). And random down-sampling analysis suggested R^2^ values decreased as the sample size decreased. And when the sample size was 10, R^2^ values for EPI (0.948) and TE (0.952) were close to that of PE (0.942) (Figs 3A and S1B). These suggested that the small sample size rather than significant variation within the group was the cause of lower R^2^ value for PE.

Several sex chromosome-linked genes, such as *XIST*, *VCX2*, *VCX3B*, *EIF1AY*, *RPS4Y1*, and *DDX3Y*, were identified among the DEGs between the ST and control blastocysts. Presumably, this is because the percentages of male and female embryos were different between the ST and control groups. In the ST group, there were 6 male and 17 female embryos, whereas in the control group, there were 14 male and 8 female embryos (Figs 1B and S2A). Supervised hierarchical clustering plots showed that the unbalanced sex ratio of the embryos resulted in sex-linked genes seemingly being expressed group specifically in the TE and EPI lineages (S2B Fig). Comparison of the expression levels of identified sex-linked DEGs in the embryos of the same gender between ICSI control group and ST group showed that although these genes tended to be expressed differentially between ST and ICSI embryos, almost all of them were not statistically significant with Bonferroni correction (S2C Fig). Significant difference between ST and ICSI groups was only seen for *XIST* in TE cells of male embryos with Log_2_(fold change) less than 1 (0.91) (S2C Fig). Simulation within TE also carried out and when the number of female embryos in the ST group was 3, these 2 groups had the closest sex ratio and the lowest ratio (6.3%) of sex-linked DEGs (S3 Table). These confirmed our hypothesis that imbalanced sex ratio was indeed the main confounding factor for the DEG analysis of the sex-linked genes.

Notably, single-cell regulatory network inference and clustering (SCENIC) analysis based on regulons also divided the cells into 3 distinct lineages, and cells of the ST blastocysts still clustered together with the same lineage of cells of ICSI control blastocysts in both the t-SNE map and the hierarchical clustering plot (Fig 3B and 3C). Specifically, the ST and control blastocysts were highly similar, if not identical, in terms of transcriptional regulatory networks.

**Fig 4:**
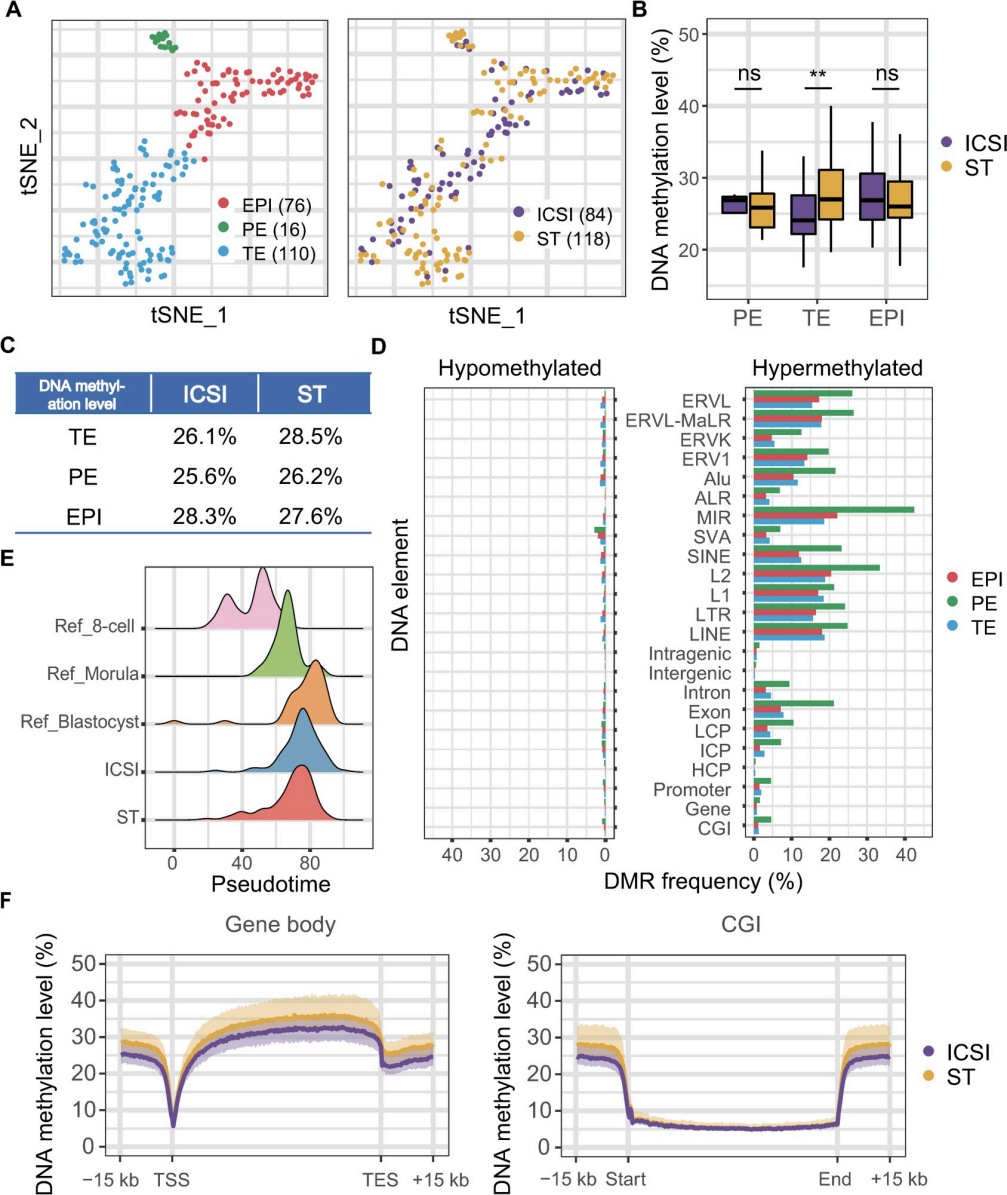
DNA methylome analysis of selected cells. (A) Two hundred and two cells remained after quality control and were projected onto a t-SNE map of their transcriptome data. The 3 lineages were clearly separated, while the ST and control groups clustered together well. (B, C) Overall DNA methylation levels for each lineage of cells. The *p*-values in the boxplot (B) were calculated by the Wilcoxon sum test (ns, *p* > 0.05; **, *p* < 0.01). The DNA methylation levels of the embryonic cells distinguished by group (column) and lineage (row) are listed in Table (C). (D) DMR analysis. Hypomethylated regions (genomic regions with DNA methylation levels low in ST cells but high in ICSI cells) and hypermethylated regions (regions with DNA methylation levels low in ICSI cells but high in ST cells) were annotated to DNA elements. The DMR frequency shows the enrichment of DMRs within DNA elements. For example, a hypermethylated DMR frequency in CGIs indicates the ratio of the number of hypermethylated DMRs located in CGIs to the total number of CGIs. (E) Ridge plot of the DNA methylation pseudotime trajectory. Cells of an earlier stage (8-cell stage, morula, blastocyst; Ref, reference) were mixed with cells in this research, and PCA was performed. The value of PC1 was interpreted as pseudotime for each cell. (F) DNA methylation levels of gene bodies and CGIs and their flanking 15-kb regions. The methylation level of each cell in the corresponding group is indicated by the transparent area around the line. The solid lines indicate the mean methylation levels of ST (purple) and ICSI (yellow) groups. The numerical data are listed in S2 Data. CGI, CpG island; DMR, differentially methylated region; EPI, epiblast; ICSI, intracytoplasmic sperm injection; PE, primitive endoderm; ST, spindle transfer; TE, trophectoderm; t-SNE, t-distributed stochastic neighbor embedding.

DNA methylation plays critical roles in the development of mammalian embryos. Using the scTrio-seq2 method, we subsequently selected 268 individual cells from 32 embryos for DNA methylome sequencing, and 217 cells from 29 embryos (132 cells from ST blastocysts and 85 cells from ICSI control blastocysts) passed quality control (S3A Fig). The t-SNE analysis and DEG analysis revealed the same conclusion that, in general, there was no difference in the transcriptomes of the same lineage of cells between the ST and ICSI control groups for cells whose DNA methylomes were also sequenced and analyzed (Figs 4A and S3E and S2 Table).

We found that the global DNA methylation levels of the cells of ST blastocysts were slightly higher than those of control embryos (Figs 4B and 4C and S3C). This indicates that global DNA demethylation in the ST embryos may be slightly delayed compared with that in the control embryos.

We further investigated the DNA methylation level in each lineage. Specifically, the DNA methylation levels of EPI and PE of ST blastocysts were comparable to those of control blastocysts, with no significant differences (27.6% versus 28.3% for EPI; 26.2% versus 25.6% for PE) (Fig 4B and 4C). Additionally, the profiles of the DNA methylation levels of the gene body and CpG islands (CGIs) were similar between these two groups (Figs 4F and S5 and S6). However, the DNA methylation level of TE cells from ST blastocysts (28.5%) was 2.4% higher than that of TE cells from control blastocysts (26.1%) (Fig 4B and 4C). We also analyzed the DNA methylation levels of different genomic elements between ST and control blastocysts. In general, there were no DNA methylation level differences in the genomic regions we analyzed in EPI or PE cells between the ST and control groups. On the other hand, for the TE lineage, the majority of genomic elements we analyzed presents methylation levels that were higher in the ST embryos than in the control embryos (S3F Fig).

Differentially methylated region (DMR) analysis revealed that 152,258 300-bp genomic tiles were hypermethylated in TE cells of the ST group compared with those of the control group. On the other hand, 121,255 300-bp genomic tiles were hypomethylated in TE cells from the ST group compared with those from the ICSI control group. Both hypermethylated and hypomethylated regions of ST blastocysts were enriched in repetitive sequences (Fig 4D). And there is no obvious clustering in the genome-wide spatial distribution of these DMRs (S4B Fig).

Since human embryos undergo a drastic global DNA demethylation process during preimplantation development, it is reasonable to speculate that the DNA demethylation process is slightly delayed in ST blastocysts, resulting in higher residual DNA methylation levels. To test this hypothesis, a pseudotime trajectory was constructed using our previously published DNA methylome data of human preimplantation embryos [27]. The DNA methylation patterns of ST blastocysts were closer to those of the earlier preimplantation developmental stage, while the DNA methylation patterns of ICSI blastocysts were very similar to those of the reference blastocysts (Fig 4E). Therefore, we conclude that, compared with ICSI control embryos, ST embryos showed a slightly delayed global demethylation process at the blastocyst stage. Moreover, we profiled the DNA methylation levels of each individual embryos or cells using sequencing data of TE and the results suggested that the DNA methylation levels of ST embryos or cells had an overall increasing trend (S4A Fig), and the delay in DNA demethylation process was not caused by abnormally high DNA methylation level of a small fraction of the embryos or a small fraction of the cells.

**Fig 5:**
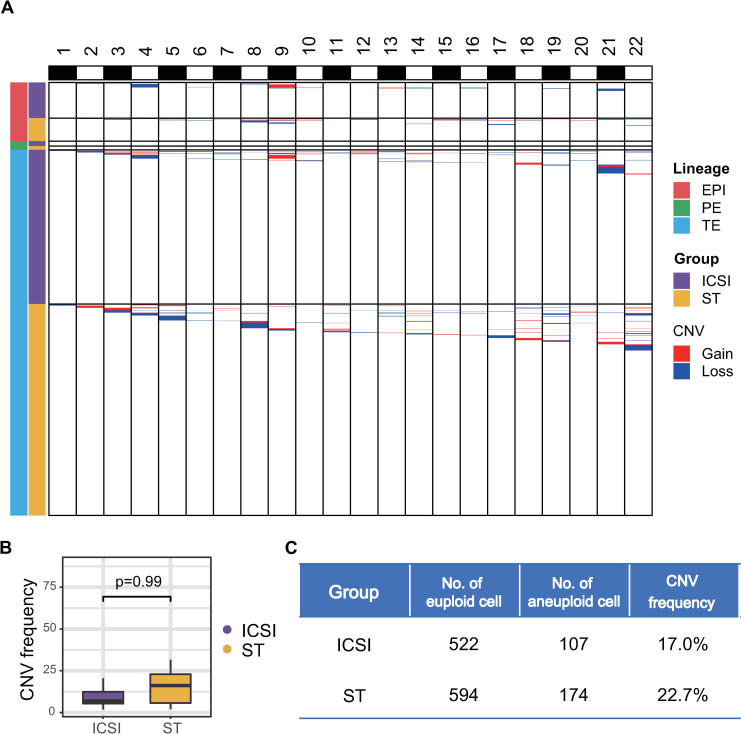
CNV analysis. (A) Global CNV patterns of the 1,397 cells from 45 embryos. (B, C) The CNV frequency for embryos was calculated based on the percentage of aneuploid cells in each embryo and tested by the Wilcoxon sum test (B). The numbers of euploid or aneuploid cells in the ST or ICSI control groups are listed in Table (C). The numerical data are listed in S2 Data. CNV, copy number variation; EPI, epiblast; ICSI, intracytoplasmic sperm injection; PE, primitive endoderm; ST, spindle transfer; TE, trophectoderm.

Next, we used the transcriptome data and previously published method [28] to infer the CNVs for each individual cell and identify aneuploid cells (Fig 5A). The reliability of the deduction was confirmed by comparison with the preimplantation genetic testing results for several of the embryos. These findings were further verified by comparison with the single-cell genome sequencing data of the same individual cell by scTrio-seq2 (S7A and S7B Fig). In general, the percentages of aneuploid cells in ST blastocysts were comparable to those in ICSI blastocysts (22.7% versus 17.0%) (Fig 5B and 5C). This suggests that ST manipulation does not increase the percentage of aneuploid cells in blastocysts.

Additionally, after the aneuploid cells were removed, the transcriptome landscapes of the euploid cells of these 3 lineages were still nearly identical between the ST and control groups, with fewer than 30 DEGs identified (S7C Fig and S2 Table), further verifying that ST manipulation in general did not affect the gene expression patterns of any of the 3 lineages of blastocyst cells.

Finally, the relationship between gene expression levels and CNVs was investigated, which suggested a positive correlation. Because the size and location of CNV varied on each chromosome, gene expression levels fluctuated between 1.04 to 1.29 when the copy number increased and between 0.92 to 0.54 when the copy number decreased (S7D Fig).

To further integrate transcriptome and DNA methylome, multiomics factor analysis (MOFA) [29] was applied to cells that had both omics data. As an unsupervised dimensionality reduction method, MOFA inferred 5 hidden factors capturing the principal sources of biological variations in multiomics data (S8A Fig). The factor 1 and factor 2 (or factor 1 and factor 5) (ranked by variance explained) together captured the characteristics of 3 cell lineages best, which could divide the cells into 3 lineages (EPI, PE, and TE), and cells from the 2 groups were still clustered together (S8C Fig). And pairwise combination of the top 5 factors could not separate the cells of ST and ICSI blastocysts (S8C Fig). Uniform manifold approximation and projection (UMAP) analysis based on these factors also divided the cells into 3 cell lineages, and cells of ST and ICSI embryos were still clustered together (S8B Fig).

## Discussion

Mitochondria are the main source of adenosine triphosphate generation in eukaryotic cells, and energy-intensive tissues are especially vulnerable to mitochondrial dysfunction. As early as 1988, studies have described diseases (mitochondrial myopathies, Leber’s hereditary optic neuropathy, and Kearns–Sell syndrome) caused by mtDNA mutations [30–32]. More than 30 years passed, and now hundreds of mitochondria mutations have been reported; there is still no effective way to eradicate mitochondrial diseases. Nowadays, limited treatments are palliative and largely based on disease symptoms, for example, insulin or hypoglycemic drugs for patients with diabetes, and cardiac electronic implantable devices for patients with heart block [33,34]. Remarkable clinical variability and progressive course contribute to the difficulty in therapy. And it is imperative for women harboring pathogenic mtDNA mutations to prevent transmission of diseases to offspring and avoid involuntary childlessness. Unlike nuclear genome mutations, for women with a high percentage of mutated mtDNAs, prenatal or preimplantation genetic diagnosis may be useless [8].

As a path of germline remediation, ST offers a glimmer of hope for families affected by mtDNA-related diseases that might have healthy children genetically related to both parents. One of the key factors driving ST to the clinic is safety. In this study, we provided systematic safety evaluations. The blastocyst stage is the last in vitro developmental stage of the assisted reproductive technology (in vitro fertilization and embryo transfer), which already set the foundation of embryonic development. Its quality is vital for successful embryo transfer and full-term delivery. So, we chose human blastocysts to do single-cell multiomics analyses. We analyzed the transcriptome, DNA methylome, and genome (CNV) landscapes of ST blastocysts and compared them systematically to those of ICSI control blastocysts at single-cell resolution. In general, the ST blastocysts were highly similar to the control embryos in terms of RNA expression, with only a few genes differentially expressed between them across all 3 lineages of cells. On the other hand, the ST blastocysts presented comparable DNA methylation levels to the control for EPI and PE, but moderately higher DNA methylation in TE. The pseudotime analysis indicated that this phenomenon is most likely due to a slightly delayed global DNA demethylation process. It is quite possible that after the blastocyst stage, ST embryos can “catch up” to complete the DNA demethylation process before implantation. Additionally, we did not observe a significant increase in the percentage of aneuploid cells in the ST blastocysts, which suggests that ST did not cause dramatic increases in aneuploidy events. Thus, ST did not disturb the genomic stability and integrity of spindle-transferred embryos. This is consistent with previous studies, but with a larger number of embryos analyzed, and single-cell sequencing allows for higher resolution and more accurate measurements [18,20].

Besides mitochondrial disease, female age-related infertility and spontaneous abortion are often associated with mitochondrial dysfunctions. In general, reproductive aging is associated with mtDNA mutations, alteration of mtDNA levels, mitochondria morphology, and dynamics in oocytes [35–37]. And de novo mitochondrial mutations accumulate as mammalian oocytes aged, which affects fertility unfavorably [38]. Therefore, ST can be extended to a wider range of clinical applications to benefit more patients, under the condition of technical safety and ethical permission [39]. Also, it will be necessary to closely monitor the postnatal development of live birth babies for longitudinal studies.

Combining the systematic and comprehensive analyses of the transcriptome, DNA methylome, and genome (CNV), we conclude that ST seems generally safe and does not severely disturb embryonic development. However, given the limitation of the study, more dimensions and larger-scale evaluations are still needed to determine whether ST can be applied to a wider set of clinical trials. First, due to the rarity of human embryos, especially ST embryos, only 46 blastocysts were included in this study, which is a relatively limited number. And only the blastocyst stage, the last in vitro developmental stage for in vitro fertilization, was analyzed by single-cell multiomics sequencing. Although it is crucial that the molecular characteristics of ST blastocysts are identical with those of ICSI blastocysts for continued assisted reproduction, the single sampling stage prevents us from assessing the presence of developmental abnormalities in ST embryos at earlier embryonic stages. And to avoid erroneous conclusions caused by poor-quality sequencing data, we filtered the cells according to the fairly stringent criteria. Therefore, the cells that were filtered out, including those damaged in experiments or stunted of their own, could not be assessed in this study. The issues abovementioned need to be solved and explored in the future. Also, many more aspects of embryonic biology, such as three-dimensional genomic structures and chromatin states, should be analyzed in ST embryos to determine whether any abnormalities occur with respect to these aspects of embryo biology in the future. In conclusion, our work provides the first comprehensive set of molecular evidences indicating that ST seems generally safe in human embryos and deserves further rigorous scientific evaluations and even clinical testing.

## Materials and methods

### Ethics statement

This study was reviewed and approved by the Medical Ethics Committee of the Sixth Medical Center of Chinese PLA General Hospital (HZKY-PJ-2019-3). A total of 30 female donors aged 25 to 42 years were recruited from the reproductive center of the Sixth Medical Center of the Chinese PLA General Hospital. All the donors provided written consent. Ovarian stimulation of oocyte donors was carried out with gonadotrophins followed by human chorionic gonadotrophin (Livzon, Zhuhai, Guangdong, China, Cat. 5,000 IU or 10,000 IU) to retrieve oocytes. Sperm were donated by the husbands of female donors who signed informed consent forms. The procedures for embryo collection, vitrification, thawing, and culture followed those of standard clinical protocols [40,41].

### Reconstructed embryos obtained by ST

ST was carried out based on previously published methods [24]. Briefly, 2.5 hours after oocyte retrieval or thawing, MII-stage oocytes were exposed to Sydney IVF Gamete Buffer (Cook Medical, Bloomington, Minnesota, USA, Cat. K-SIGB-20) with 7.5 μg/mL cytochalasin B (CB) (Sigma-Aldrich, Saint Louis, Missouri, USA, Cat. 250233) for 5 minutes at 37°C. Subsequent manipulation was performed under an inverted microscope (Olympus, Shinjuku, Tokyo, Japan, Cat. IX-73) equipped with micromanipulators and a warm stage. The zona pellucida next to the spindle was then drilled using the Saturn Active Laser System (RI Research instruments, Mainz, Rheinland-Pfalz, Germany, Cat. 6-47-500) with several pulses of 100 to200 μs, which allowed the spindle to be gently aspirated into the pipette. The spindle was immediately transferred into the perivitelline space of an enucleated recipient oocyte. The reconstructed oocytes were transferred into CB-free Gamete Buffer and incubated for 10 minutes at 37°C.

Fusion between the spindle and the enucleated recipient oocyte was initiated by an electric pulse. We placed the unfused reconstructed oocyte into BTXpress Cytofusion Medium C (BTX, Holliston, Massachusetts, USA, Cat. 47–0001) between platinum electrodes and applied a single electrical pulse (5 V AC for 2 seconds, 1.3 kV/cm DC for 35 μs) using an electro cell fusion system (BEX, Itabashi, Tokyo, Japan, Cat. CFB16-HB) at room temperature. The reconstructed oocytes were then washed for 3 times and cultured in Sydney IVF fertilization medium (Cook Medical, Cat. K-SIFM-20) at 37°C in 6% CO_2_ and 5% O_2_ for 30 minutes to check their fusion status.

Next, each reconstructed oocyte was fertilized by ICSI with a single immobilized sperm of normal morphology and then cultured in Sydney IVF cleavage medium (Cook Medical, Cat. K-SICM-20) for 2 days in 6% CO_2_ and 5% O_2_ at 37°C and then in Sydney IVF blastocyst medium (Cook Medical, Cat. K-SIBM-20), which was replenished every 48 hours until blastocyst formation occurred.

### Collection of human embryos and individual cells

Both ST blastocysts and ICSI control blastocysts were collected on days 5 to 7 postfertilization, and no delayed or arrested embryos were included in this study. We first treated the blastocysts with acidic Tyrode’s solution (Sigma-Aldrich, Cat. T1788) using a mouth pipette to remove the zona. The blastocysts were then transferred to digestion medium (Accutase medium: 0.25% trypsin, 1:1) (Sigma-Aldrich, Cat. A6064; Gibco, Carlsbad, California, USA, Cat. 25300–056) drops at 37°C for 10 to 20 minutes with gentle and careful pipetting to isolate individual cells. The inner diameters of the glass pipettes varied from 10 to 30 μm. Individual cells were selected and collected into 200 μL PCR tubes with lysis media. All the procedures were performed under a stereomicroscope (Nikon, Shinagawa, Tokyo, Japan, Cat. SMZ745).

### Single-cell Trio-seq2 sequencing

Single-cell Trio-seq2 sequencing libraries were prepared based on a previously published method, with slight modifications [25]. Briefly, a single cell was picked into a 200-μL PCR tube containing lysis buffer with 0.2 μL of magnetic beads (Invitrogen, Carlsbad, California, USA, Cat. 65011). mRNA and cell nuclei were separated to construct libraries of transcriptomes and DNA methylomes, respectively.

For scRNA-seq libraries, the supernatant was transferred to a new 200-μL PCR tube. First, we reverse transcribed the mRNAs to obtain first-strand cDNAs using SuperScript II Reverse Transcriptase (Invitrogen, Cat. 18064071) and designed primers that contained an oligo(dT) primer sequence, a cell-specific barcode, and unique molecular identifiers (UMIs). Next, double-stranded cDNA was synthesized and amplified. After pooling and purification of the samples with different barcodes, the cDNAs were amplified using a primer anchored with biotin and index tags and then fragmented into fragments of approximately 300 bp by a Covaris S2 sonicator (Covaris, Woburn, Massachusetts, USA, Cat. S220). After enriching the cDNA fragments with Dynabeads MyOne Streptavidin C1 beads (Invitrogen, Cat. 65002), sequencing libraries were subsequently generated using Kapa Hyper Prep Kits (KAPA Biosystems, Boston, Massachusetts, USA, Cat. KK8504).

The post-bisulfite adaptor tagging (PBAT) strategy was used to generate single-cell DNA methylome libraries. Genomic DNA was released after proteinase K (NEB, Ipswich, Massachusetts, USA, Cat. P8107S) treatment, and bisulfite conversion (Zymo Research, Irvine, California, USA, Cat. D5044) was carried out. After bead-based purification, DNA was complemented with Klenow polymerase (NEB, Cat. M0210L) and the random primer P5-N6, and this step was repeated 3 times. A P7-N6 random primer was then used to synthesize the second strand, and the final libraries were constructed after PCR amplification.

All library sequencing was performed on an Illumina HiSeq 4000 platform or an Illumina NovaSeq platform (Novogene, Beijing, China) in paired-end 150-bp mode.

### Single-cell RNA-seq data preprocessing

Paired-end reads with cell barcodes located in the first 8 bp of read 2 were extracted (via zero tolerance) with UMI-tools (v0.5.4). The sequences of the cell barcode and UMI (CCCCCCCCNNNNNNNN (C for cell barcode and N for UMI)) were simultaneously attached to the read names of read 1 and read 2 for subsequent demultiplexing. Template-switching oligos (TSOs) and poly-A sequences were trimmed via a custom-made Perl script and the “trimfq” command of seqtk (v1.0-r82-dirty), respectively. The clean reads obtained above were then aligned to the reference genome GRCh38-1.2.0 by STAR (v2.6.0a) with “—outFilterMultimapNmax 1” and other default parameters. FeatureCounts (v1.6.2) assigned the aligned reads to genes, and then the UMI-tools (v0.5.4) count command was used to generate the UMI count matrix. Low-quality cells were filtered according to their expression. We observed that, with 4,000 as the limit, the number of genes per cell clearly showed 2 distributions. After careful further investigation, we found that the cells expressing fewer than 4,000 genes were considered poor-quality TE cells. Considering that we already had enough TE cells and that EPI and PE cells play more important roles in embryonic development, we excluded cells in which fewer than 4,000 genes are expressed from subsequent analysis. The UMI counts of genes were normalized by ln(TPM/10+1). Only genes with a ln(TPM/10+1) expression greater than 1 by more than 3 cells were retained for subsequent analysis.

### Identification of cell types and lineages

To identify different lineages of cells in ICSI and ST blastocysts, we first performed linear dimensional reduction (principal component analysis (PCA)) and then nonlinear dimensional reduction (t-SNE and UMAP) with the “Seurat” package (v4.0.0) on the filtered expression matrix. After partitioning cells into clusters in the t-SNE plot, high-confidence markers (*GATA3*, *GATA2*, and *CDX2* for the TE lineage; *GATA4*, *FOXA2*, and *SOX17* for the PE lineage; and *SOX2*, *NANOG*, and *POU5F1* for the EPI lineage) were used to identify different lineages of cells. To further verify the accuracy of lineage identification, we identified lineage-specific signatures using the “FindAllMarkers” function of the “Seurat” package (v4.0.0) with the parameters ident.1 = “ST,” ident.2 = “ICSI,” min.pct = 0.25, and test.use = “wilcox” and compared them with commonly used EPI, PE, and TE markers. The identified lineage-specific signatures were further used to calculate lineage scores as previously described [25]. Briefly, the lineage scores were calculated for each cell as the mean expression levels of the lineage-specific signatures, which were then normalized to a range of 1 to 100 and visualized via the “ggtern” package (v3.3.0).

### Comparative analysis of gene expression patterns

To investigate the differences in expression levels between the ST and control blastocysts, the mean expression level (ln(TPM/10+1)) of each gene was calculated for each lineage and each group to obtain global expression patterns. The correlation coefficient of the global expression pattern between the ICSI and ST blastocyst cells was then calculated via linear regression analysis. The same method was applied when the cells from TE or EPI lineage were randomly down-sampled for investigating the effect of sample size to abovementioned correlation coefficients. Genes that were differentially expressed between the cells of ICSI and ST blastocysts were identified using the “FindMarker” function in the “Seurat” package (v4.0.0) with the parameters ident.1 = “ST,” ident.2 = “ICSI,” min.pct = 0.25, and test.use = “wilcox” for each lineage. Only DEGs with an average log_2_(fold-change) greater than 1 and an adjusted *p*-value (based on Bonferroni correction) less than 0.01 were characterized as up-regulated DEGs, and only those with an average log_2_(fold change) less than −1 and an adjusted *p*-value (based on Bonferroni correction) less than 0.01 were characterized as down-regulated DEGs. For those sex chromosome-linked genes, we further explored their detailed expression patterns by the “FindMarker” function with the parameters ident.1 = “ST,” ident.2 = “ICSI,” min.pct = 0, and test.use = “wilcox” but restricted the cells to be in the same lineage and the same gender. The adjusted *p*-values were also calculated based on Bonferroni correction.

### Inference of embryo sex

To infer the sex of each embryo, we used a previously published method [25]. Specifically, we identified X-linked and Y-linked genes and calculated the mean expression levels of them for each embryo, yielding Mean_X and Mean_Y, respectively. The sex of the embryos was then determined by the ratio of Mean_X to Mean_Y. According to previous experience, embryos with Mean_X/Mean_Y >2 should be female, and embryos with Mean_X/Mean_Y≤2 should be male. We verified the results of this analysis by analyzing the CNV of single-cell DNA methylation sequencing (PBAT-seq) data.

### SCENIC analysis

We used the “SCENIC” package (v1.2.4) to analyze the gene regulatory network for the scRNA-seq data. The inference of the transcription factor-driven regulatory network was performed by pySCENIC (0.11.0), a lightning-fast Python implementation in the SCENIC pipeline. Briefly, the input expression matrix in loom format was prepared with the “SCopeLoomR” package (v0.10.4). A coexpression network was subsequently constructed with the embedded GRNBoost2 package. The embedded RcisTarget package was used to calculate the enriched motif and corresponding target genes for all modules gathered by GRNBoost2. Regulons were generated with centered transcription factors and target genes. Regulon enrichment was measured via the embedded AUCell package. We used the output AUC score to perform t-SNE via the “Rtsne” package (v0.15) and hierarchy clustering via the “hclust” function in the “stats” package (v4.0.3), with the parameters distance = Euclidean and method = ward.D2, respectively.

### Single-cell PBAT DNA methylome sequencing data preprocessing

First, trim_galore (v0.6.4) was used to trim the primers and low-quality bases, with the parameters—quality 20—stringency 3—length 50—clip_R1 6—clip_R2 6—paired—phred33. The sequences of the clean reads obtained above were then mapped to the human genome (hg38) by Bismark (v0.22.3) with embedded Bowtie2 (v2.3.5) in paired-end and nondirectional modes. The sequences of unmapped reads were obtained and realigned to the reference genome hg38 by Bismark (v0.22.3) in single-end mode. All the mapped reads were merged and sorted by coordinates with SAMtools (v1.10), and all PCR duplicates were removed with the “rmdup” command in SAMtools. The conversion ratio was calculated from the unmethylated level of lambda DNA. Samples with lambda conversion ratios greater than 99% and covering at least 2e+6 CpG sites with at least 1 read for each CpG site were considered to have passed quality control. The DNA methylation level at each site was calculated with MethylDackel (v0.5.1). Specifically, the methylation level at each site was calculated by dividing the number of reads supporting C (methylated) by the total number of reads supporting C (methylated) and T (unmethylated). The DNA methylation level per cell was calculated from the overall mean methylation level of the CpG sites. And the DNA methylation level per embryo was calculated from the mean DNA methylation level of the cells.

### DMR analysis

The BAM files for all the cells of the same group and the same lineage were merged with the “merge” function of SAMtools (v1.10). We then applied a 300-bp tile-based DNA methylation calling algorithm with a custom-made Perl script as previously described [27]. Briefly, we binned the genome to consecutive 300-bp tiles and interpreted the ratio of the number of reads supporting C to the total number of reads supporting C and T in the 300-bp tiles as the DNA methylation level of the 300-bp tiles. Those 300-bp tiles with DNA methylation levels less than 10% in 1 group and greater than 40% in the other group were defined as DMRs. To further annotate these DMRs, we compared these DMRs with specific genetic elements by the “intersect” function of BEDtools (v2.29.0). Spatial distribution of DMRs were plotted by the function “kpPlotRegions” of the R package karyoploteR (v1.18.0).

### Pseudotime analysis of DNA methylome data

Early-stage data were retrieved from a previously published paper [27] and preprocessed via the same pipeline as documented above. We executed a 300-bp tile-based DNA methylation calling algorithm on all the samples in this research and reference. Common tiles covered by at least 1 sample were collected and formed into a tile matrix. PCA was subsequently used to extract the main principal components from the tile matrix. The first principal component (PC1) was interpreted as the pseudotime.

### Inference of CNVs from scRNA-seq data

CNV data were inferred via the “inferCNV” package (v1.8.1) according to a previously published method [28]. In brief, the CNV score for each gene was defined as the mean expression level of 50 upstream genes and 50 downstream genes. The CNV score for each gene was then normalized to the mean CNV score of all the cells. To further denoise the inferCNV output and to avoid sampling bias, only CNVs that exceeded 60% of the length of the chromosome for at least 3 cells were considered CNVs for that particular chromosome in subsequent analysis. Moreover, only CNVs supported by at least 3 cells were counted as CNVs of that embryo. Cells without CNVs were characterized as euploid; those with CNVs were characterized as aneuploid. The CNV frequency was derived from the percentage of aneuploid cells for each embryo.

### Integration of CNVs and RNA expression levels

We also integrated the normalized expression levels in the output of “inferCNV” and inferred CNVs for each cell to analyze their relationships. The copy number of 2 for a chromosome was treated as normal as usual. It was defined to be gain for a chromosome of a cell with copy numbers greater than 2. Otherwise, a chromosome with chromosome number less than 2 in a cell was defined as loss of a chromosome. The normalized expression levels for chromosomes with gain or loss were further normalized by the mean levels of the normal chromosomes and the normalized expression levels for normal chromosomes were also divided by their own mean levels to get the baseline.

### Inference of CNVs from scPBAT-seq data

We inferred CNVs from single-cell DNA methylation data to verify our CNV results obtained from scRNA-seq data. First, we binned the genome (hg38) into consecutive 1-M-bp windows and counted the reads reported in each 1-M-bp window. Next, poor-quality cells whose minimum median read was less than 500 were filtered and removed, and low-abundance windows whose minimum median read was less than 500 were filtered and removed. After quality control, each cell was normalized by the total number of reported reads to avoid sample size bias, and each window was normalized by the median number of reads across all the cells or selected control cells. Embryo E23 was selected as a control because we observed no CNVs in any of the cells of this embryo. Smoothing was further executed on the normalized CNV matrix. For each cell, each window was replaced with the nearest integer of the average signals of the surrounding 100 windows.

### Integration of transcriptome and DNA methylome data

We integrated the result of transcriptome and DNA methylome by the multiomics factor analysis (MOFA2) (v1.2.2). Cells both passing the quality control of scRNA-seq data preprocessing and scPBAT-seq data preprocessing were used and grouped into ICSI or ST group. DNA methylation levels for genes passing the quality control of scRNA-seq data preprocessing were calculated based on the mean DNA methylation levels of the CpG sites located in the promoters of those genes. The promoter of a gene was region expanding from the 1,500-bp upstream to the 500-bp downstream of its TSS. After the gene to cell matrixes of transcriptome and DNA methylome had been readily prepared as the input, the function “prepare_mofa” function of MOFA was used with default settings to generate the MOFA object and the “run_mofa” function performed integration on the MOFA object by the parameter “use_basilisk = T”. Factors were ordered by their proportion of explaining the variance and the top 5 factors were used to particularize cells in the low-dimension space. UMAP analysis was further performed with all of the factors.

## Supporting information

S1 Fig Comparative analysis of gene expression patterns by cell lineages. (A) Volcano plots of DEGs across lineages. Up-regulated DEGs in the ST group were defined according to a log_2_(fold change)> 1 and an adjusted *p*-value < 0.01. Down-regulated DEGs were defined according to a log_2_(fold change) <−1 and an adjusted *p*-value < 0.01. The *p*-values calculated by the Wilcoxon test were adjusted by Bonferroni correction. The 3 most significantly up- or down-regulated DEGs for each lineage were labeled in the corresponding volcano plot. (B) Gene expression correlations of cells committed to the same lineage in both groups. “Sample size” represents the number of cells to be randomly sampled from TE or EPI lineage. (C) Variance of genes’ expression levels by lineage and group. The numerical data are listed in S3 Data. DEG, differentially expressed gene; EPI, epiblast; ICSI, intracytoplasmic sperm injection; PE, primitive endoderm; ST, spindle transfer; TE, trophectoderm.
(TIF)

S2 Fig Inference of the sex of each embryo and analysis of sex-linked DEGs. (A) The mean expression levels of X (mean_X, pink)- or Y-linked (mean_Y, blue) genes were calculated for each embryo. Embryos with mean_X/Mean_Y >2 were considered as female; otherwise, they were considered as male. (B) Heatmaps of DEGs for corresponding lineages. X (pink)- or Y-(blue)-linked genes clustered together and are highlighted. (C) Selected X- or Y-linked DEGs were recalculated by comparing the cells from the same lineage and the same gender. The numerical data are listed in S3 Data. DEG, differentially expressed gene; EPI, epiblast; ICSI, intracytoplasmic sperm injection; ST, spindle transfer; TE, trophectoderm.
(TIF)

S3 Fig Quality control of the scMethylome-seq data. (A) Summary of the number of selected cells and embryos of distinct lineages in the ST and ICSI control groups for DNA methylation sequencing. (B) The median number of covered CpG sites (M, million) for cells in the ST and ICSI control groups, with each CpG covered by a different number of reads (depth). (C) DNA methylation levels of CpG sites in cells in the ST and ICSI groups at different depths. (D) The Wilcoxon sum test was used to calculate the significance (ns, *p* > 0.05) of the number of covered CpG sites in ST and ICSI cells. (E) Analysis of DEGs and linear regression analysis of selected cells for DNA methylome sequencing. (F) DNA methylation levels of different DNA elements in different lineages. The *p*-values were calculated by the Wilcoxon sum test (ns, *p* > 0.05; *, 0.01 < *p* < 0.05; **, 0.001 < *p* < 0.01; ***, 0.0001 < *p* < 0.001; ****, *p* < 0.0001). The numerical data are listed in S3 Data. DEG, differentially expressed gene; EPI, epiblast; ICSI, intracytoplasmic sperm injection; PE, primitive endoderm; QC, quality control; ST, spindle transfer; TE, trophectoderm.
(TIF)

S4 Fig Embryo-level or cell-level DNA methylation levels and spatial distribution of DMRs. (A) The DNA methylation levels of cells from the TE lineage were shown in embryo or cell. Embryos (or cells) from ICSI (purple) or ST (yellow) group were aligned together sharing the same x-axis. (B) Spatial distribution of DMRs in lineage. The numerical data are listed in S3 Data. DMR, differentially methylated region; EPI, epiblast; ICSI, intracytoplasmic sperm injection; PE, primitive endoderm; ST, spindle transfer; TE, trophectoderm.
(TIF)

S5 Fig Profiles of the DNA methylation levels of gene bodies and flanking 15-kb regions in cells in each embryo. The numerical data are listed in S4 Data. ICSI, intracytoplasmic sperm injection; ST, spindle transfer.
(TIF)

S6 Fig Profiles of the DNA methylation levels of CGIs and flanking 15-kb regions in cells in each embryo. The numerical data are listed in S4 Data. CGI, CpG island; ICSI, intracytoplasmic sperm injection; ST, spindle transfer.
(TIF)

S7 Fig Integrative analysis of CNVs and comparative analysis of DEGs in euploid embryonic cells. (A, B) Embryo E34 was selected to show that CNVs inferred from RNA expression levels (heatmap, A) and CNVs called from DNA methylome (dot plot, B) were consistent and can be used to validate each other. (C) Analysis of DEGs and linear regression analysis of euploid cells. (D) CNVs inferred from RNA expression levels were correlated across all of the chromosomes that comparing to the baseline (chromosomes with no CNVs, normal, green), chromosomes gaining copy numbers (red) have relative higher normalized expression levels while chromosomes losing copy numbers (blue) have relatively lower normalized expression levels. The numerical data are listed in S4 Data. CNV, copy number variation; DEG, differentially expressed gene; EPI, epiblast; ICSI, intracytoplasmic sperm injection; PE, primitive endoderm; ST, spindle transfer; TE, trophectoderm.
(TIF)

S8 Fig Integrative analysis of transcriptome and DNA methylome by MOFA2. (A) Factors were calculated from integrating transcriptome (RNA, right panel) and DNA methylome (DNA_meth, left pane) by MOFA2 and ordered by their explaining variance (Var). (B) Cells were embedded into the low-dimensional space by UMAP and colored according to the cell lineage or group they belong to. (C) Combination of the top 5 factors explaining variance to visually show the separation of cells under the consideration of cell lineage or group. The numerical data are listed in S4 Data. EPI, epiblast; ICSI, intracytoplasmic sperm injection; PE, primitive endoderm; ST, spindle transfer; TE, trophectoderm; UMAP, uniform manifold approximation and projection.
(TIF)

S1 Table Sample summary table for scTrio-seq2. (XLSX)

S2 Table List of DEGs related to Figs 3A and S3E and S7C. (XLSX)

S3 Table Simulation within TE to identify DEGs under different sex ratios. (XLSX)

S1 Data The individual numerical values in Figs 1C–1E and 2A–2C. (XLSX)

S2 Data The individual numerical values in Figs 3A–3C, 4A, 4B, 4D-4F, 5A, and 5B. (XLSX)

S3 Data The individual numerical values in S1A–S1C, S2A–S2C, S3B–S3F, and S4A and S4B Figs. (XLSX)

S4 Data The individual numerical values in S5, S6, S7A–S7D, and S8A–S8C Figs. (XLSX)

## Abbreviations

CGI: CpG island
CNV: copy number variation
DEG: differentially expressed gene
DMR: differentially methylated region
EPI: epiblast
ESC: embryonic stem cell
ICSI: intracytoplasmic sperm injection
MOFA: multiomics factor analysis
mtDNA: mitochondrial DNA
PBAT: post-bisulfite adaptor tagging
PCA: principal component analysis
PE: primitive endoderm
SCENIC: single-cell regulatory network inference and clustering
ST: spindle transfer
TE: trophectoderm
t-SNE: t-distributed stochastic neighbor embedding
TSO: template-switching oligo
UMAP: uniform manifold approximation and projection
UMI: unique molecular identifier
