# Supplementary figures and images for: Single-cell multiomics analyses of spindle-transferred human embryos suggest a mostly normal embryonic development
Source: PLoS Biol. 2022 Aug 16;20(8):e3001741. doi: 10.1371/journal.pbio.3001741 (PMC9380953; doi:10.1371/journal.pbio.3001741)

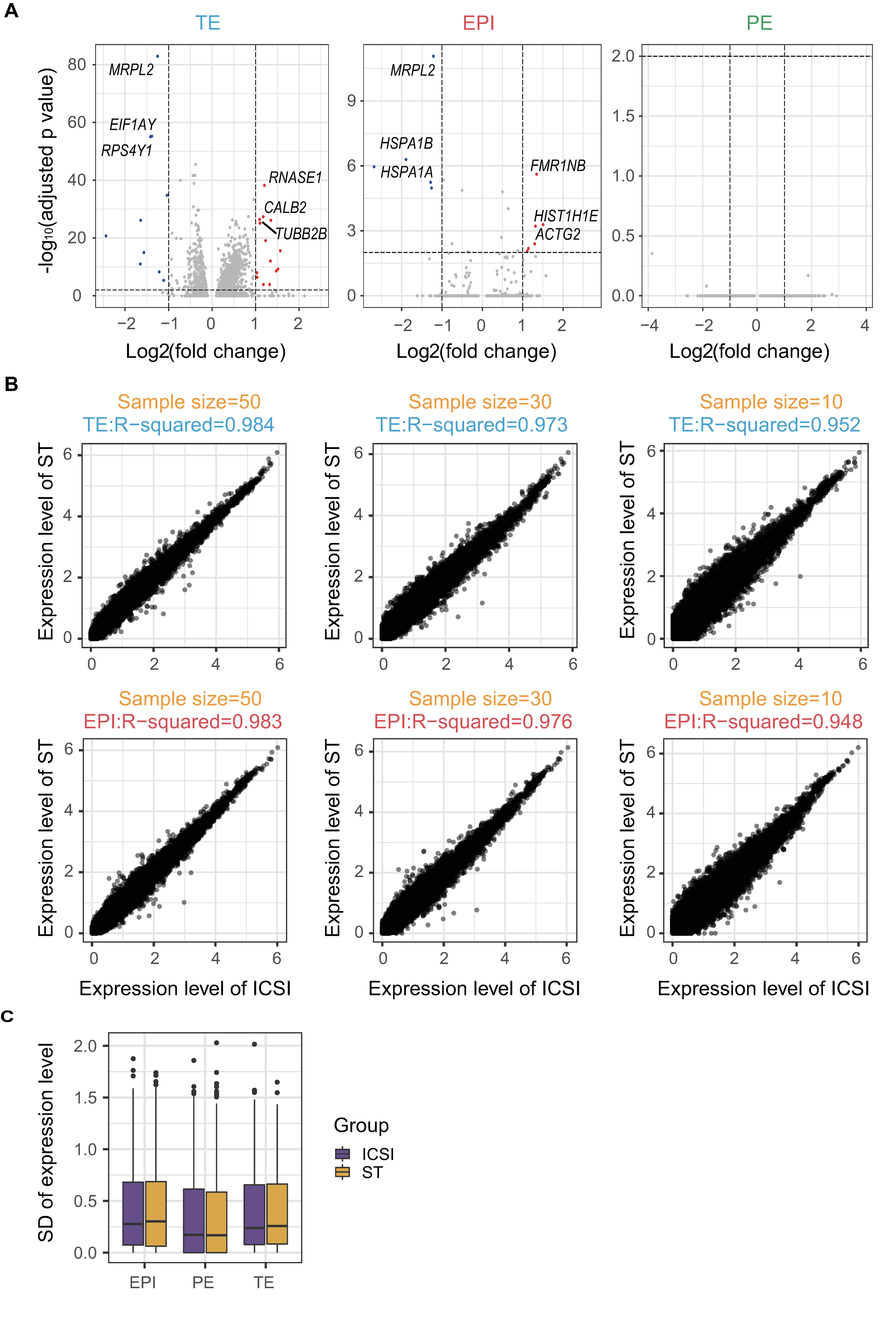

Supplement: S1 Fig — (A) Volcano plots of DEGs across lineages. Up-regulated DEGs in the ST group were defined according to a log2(fold change)> 1 and an adjusted p-value < 0.01. Down-regulated DEGs were defined according to a log2(fold change) <−1 and an adjusted p-value < 0.01. The p-values calculated by the Wilcoxon test were adjusted by Bonferroni correction. The 3 most significantly up- or down-regulated DEGs for each lineage were labeled in the corresponding volcano plot. (B) Gene expression correlations of cells committed to the same lineage in both groups. “Sample size” represents the number of cells to be randomly sampled from TE or EPI lineage. (C) Variance of genes’ expression levels by lineage and group. The numerical data are listed in S3 Data. DEG, differentially expressed gene; EPI, epiblast; ICSI, intracytoplasmic sperm injection; PE, primitive endoderm; ST, spindle transfer; TE, trophectoderm. (TIF) [file pbio.3001741.s001.tif]

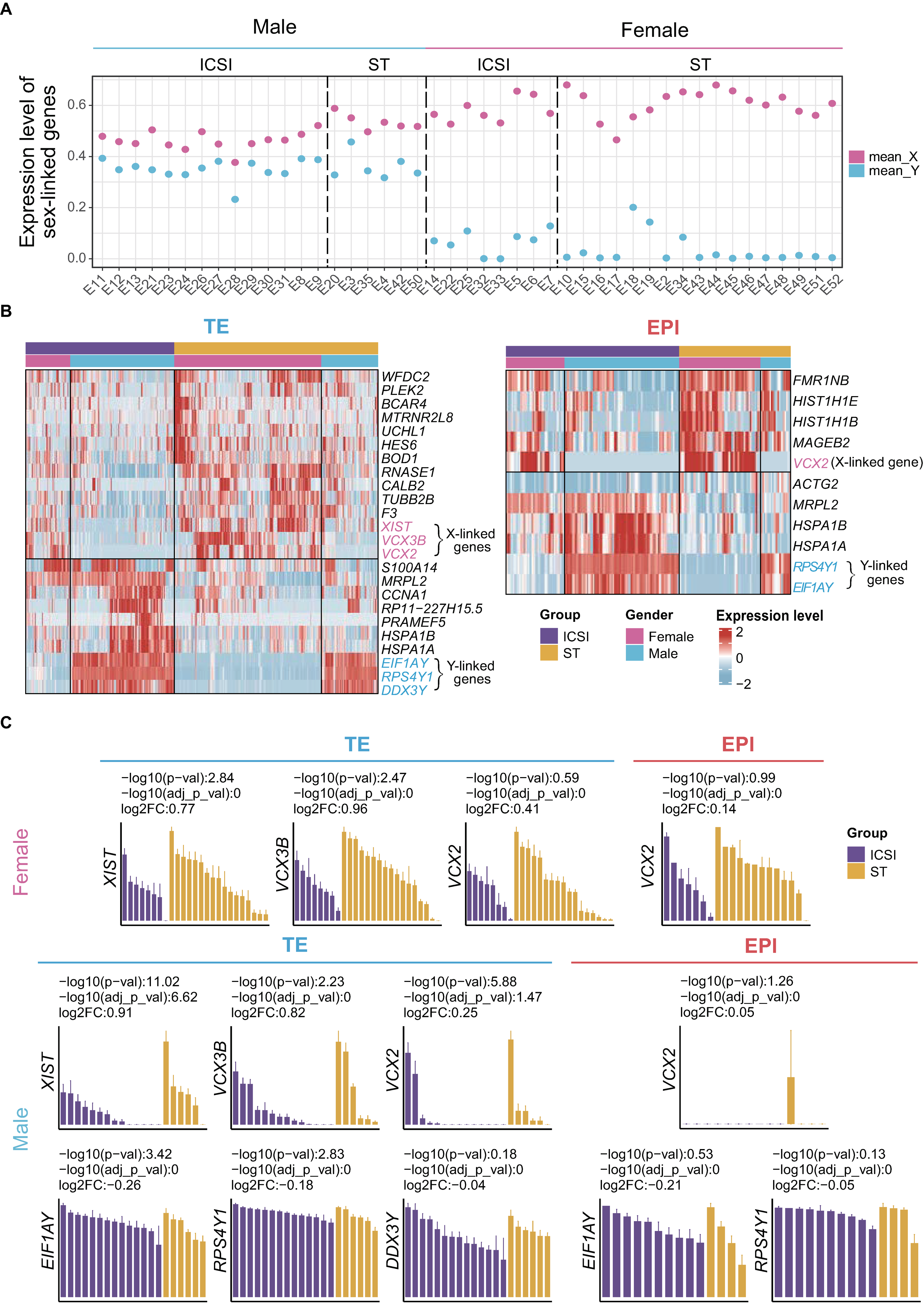

Supplement: S2 Fig — (A) The mean expression levels of X (mean_X, pink)- or Y-linked (mean_Y, blue) genes were calculated for each embryo. Embryos with mean_X/Mean_Y >2 were considered as female; otherwise, they were considered as male. (B) Heatmaps of DEGs for corresponding lineages. X (pink)- or Y-(blue)-linked genes clustered together and are highlighted. (C) Selected X- or Y-linked DEGs were recalculated by comparing the cells from the same lineage and the same gender. The numerical data are listed in S3 Data. DEG, differentially expressed gene; EPI, epiblast; ICSI, intracytoplasmic sperm injection; ST, spindle transfer; TE, trophectoderm. (TIF) [file pbio.3001741.s002.tif]

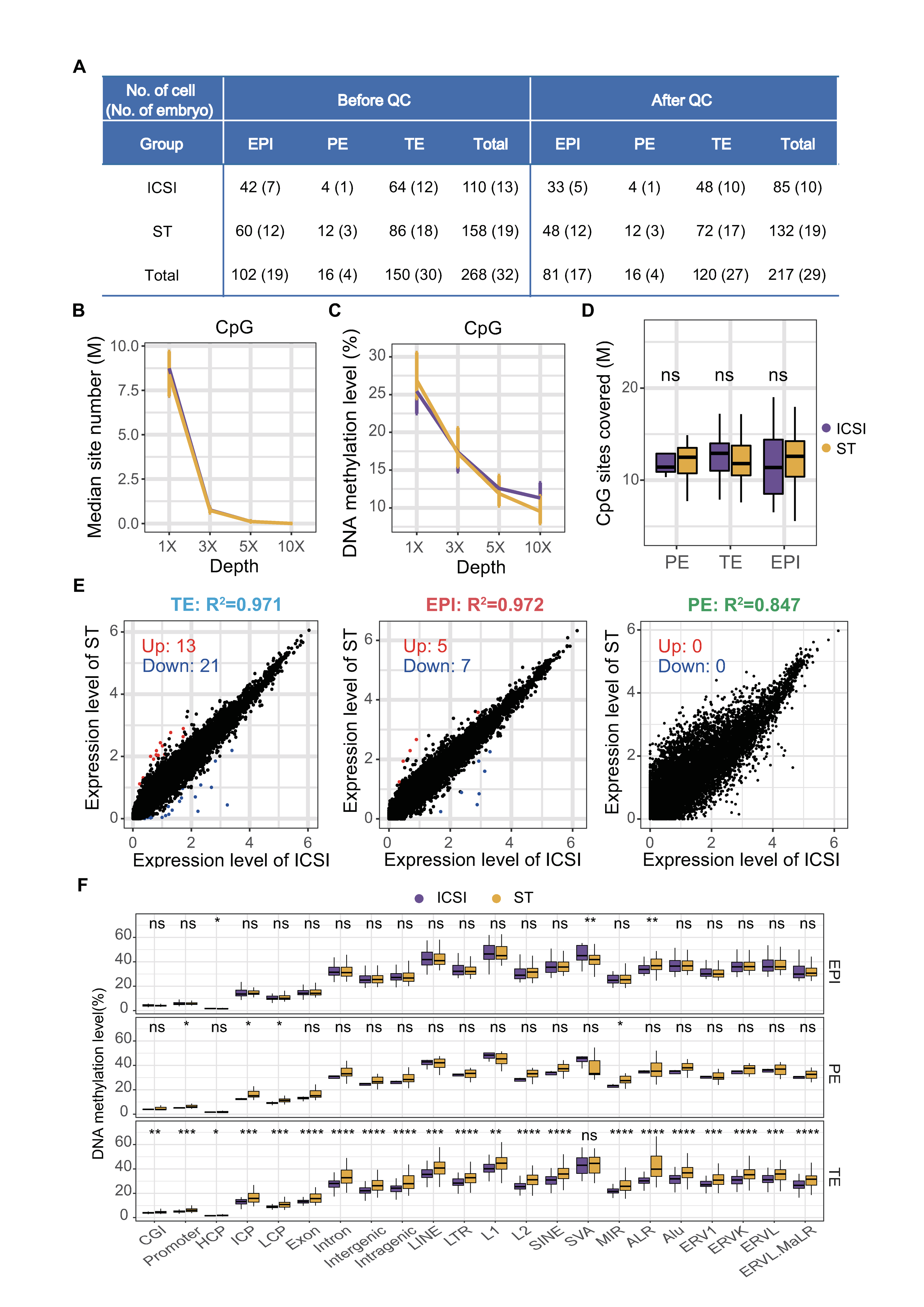

Supplement: S3 Fig — (A) Summary of the number of selected cells and embryos of distinct lineages in the ST and ICSI control groups for DNA methylation sequencing. (B) The median number of covered CpG sites (M, million) for cells in the ST and ICSI control groups, with each CpG covered by a different number of reads (depth). (C) DNA methylation levels of CpG sites in cells in the ST and ICSI groups at different depths. (D) The Wilcoxon sum test was used to calculate the significance (ns, p > 0.05) of the number of covered CpG sites in ST and ICSI cells. (E) Analysis of DEGs and linear regression analysis of selected cells for DNA methylome sequencing. (F) DNA methylation levels of different DNA elements in different lineages. The p-values were calculated by the Wilcoxon sum test (ns, p > 0.05; *, 0.01 < p < 0.05; **, 0.001 < p < 0.01; ***, 0.0001 < p < 0.001; ****, p < 0.0001). The numerical data are listed in S3 Data. DEG, differentially expressed gene; EPI, epiblast; ICSI, intracytoplasmic sperm injection; PE, primitive endoderm; QC, quality control; ST, spindle transfer; TE, trophectoderm. (TIF) [file pbio.3001741.s003.tif]

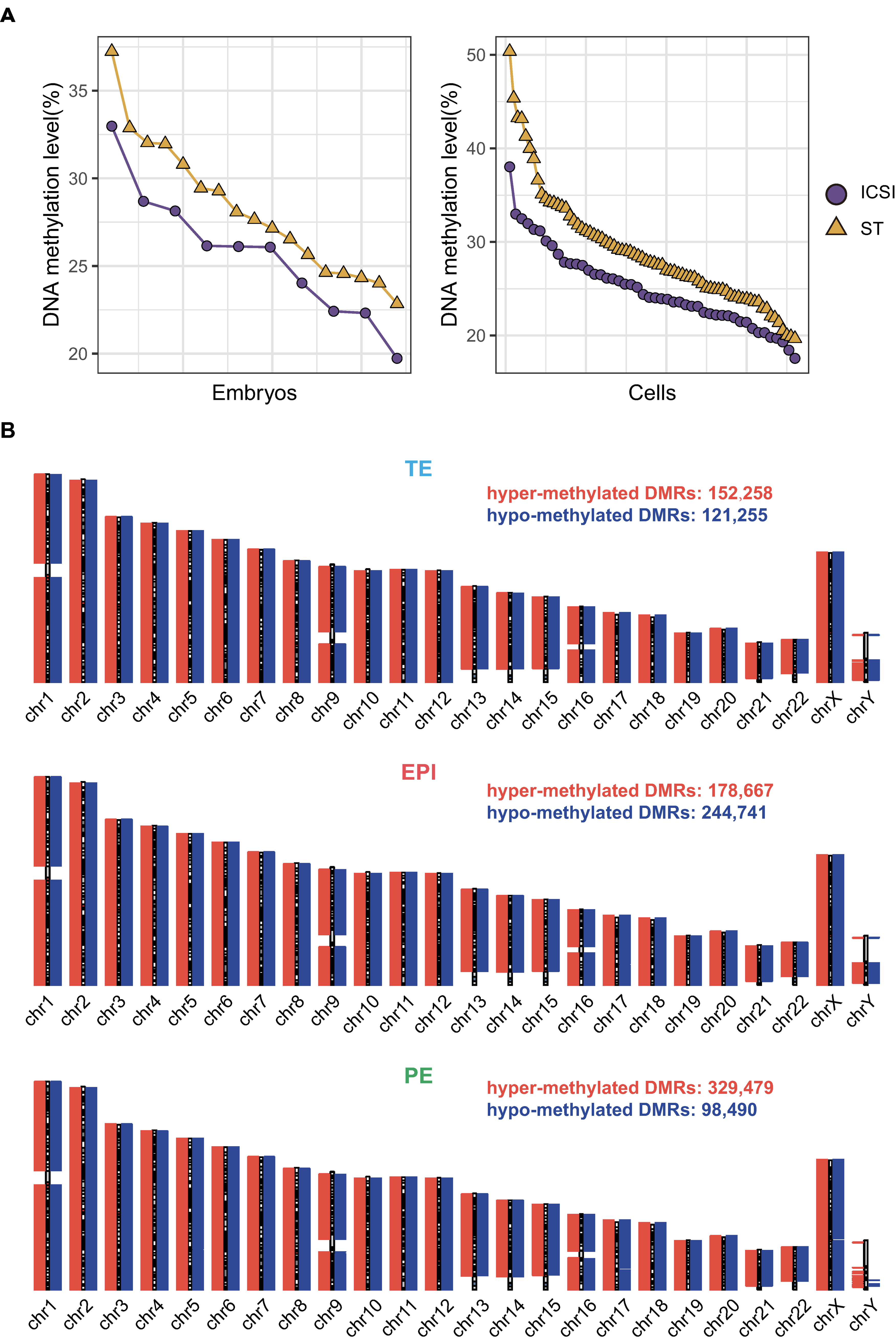

Supplement: S4 Fig — (A) The DNA methylation levels of cells from the TE lineage were shown in embryo or cell. Embryos (or cells) from ICSI (purple) or ST (yellow) group were aligned together sharing the same x-axis. (B) Spatial distribution of DMRs in lineage. The numerical data are listed in S3 Data. DMR, differentially methylated region; EPI, epiblast; ICSI, intracytoplasmic sperm injection; PE, primitive endoderm; ST, spindle transfer; TE, trophectoderm. (TIF) [file pbio.3001741.s004.tif]

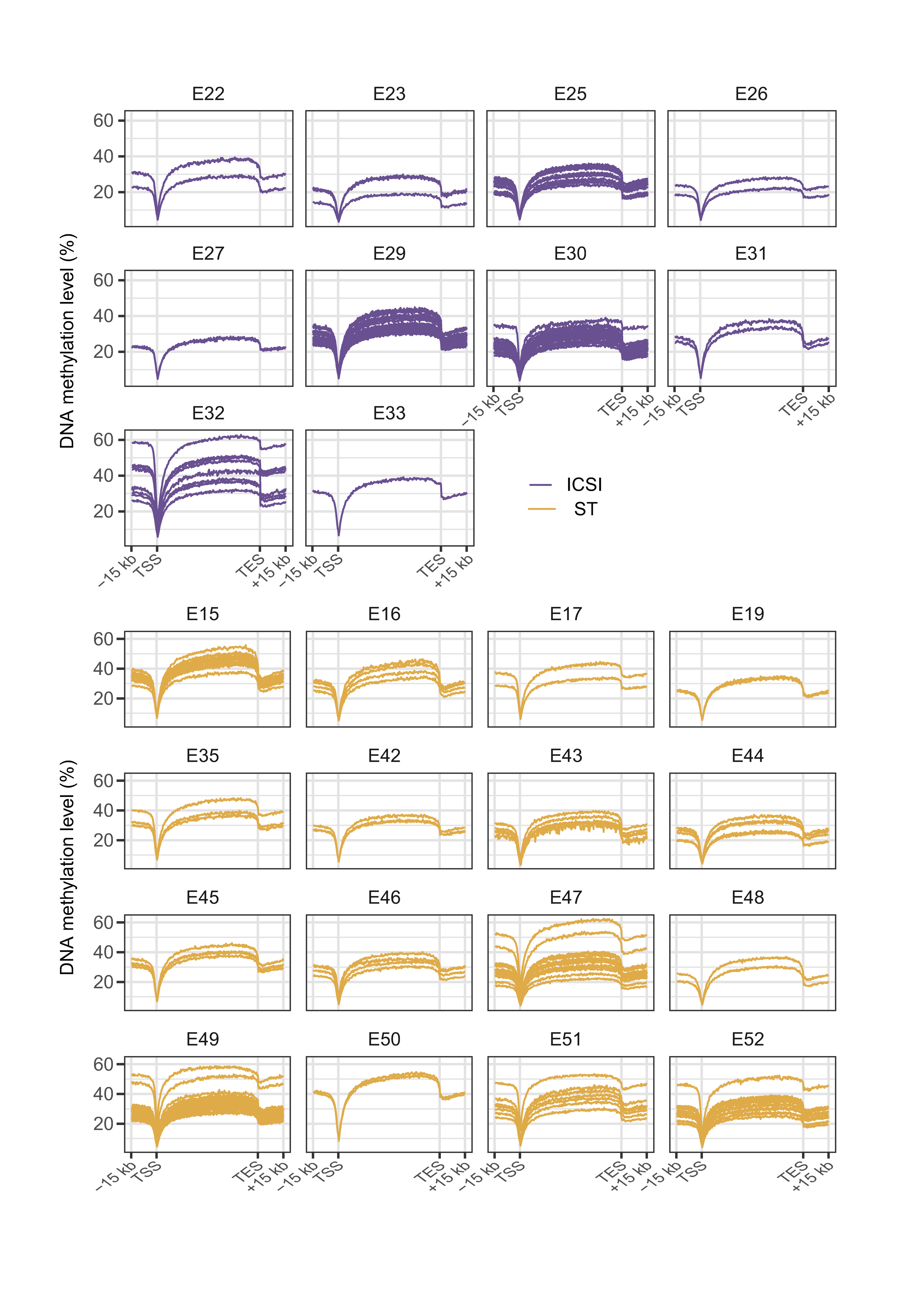

Supplement: S5 Fig — The numerical data are listed in S4 Data. ICSI, intracytoplasmic sperm injection; ST, spindle transfer. (TIF) [file pbio.3001741.s005.tif]

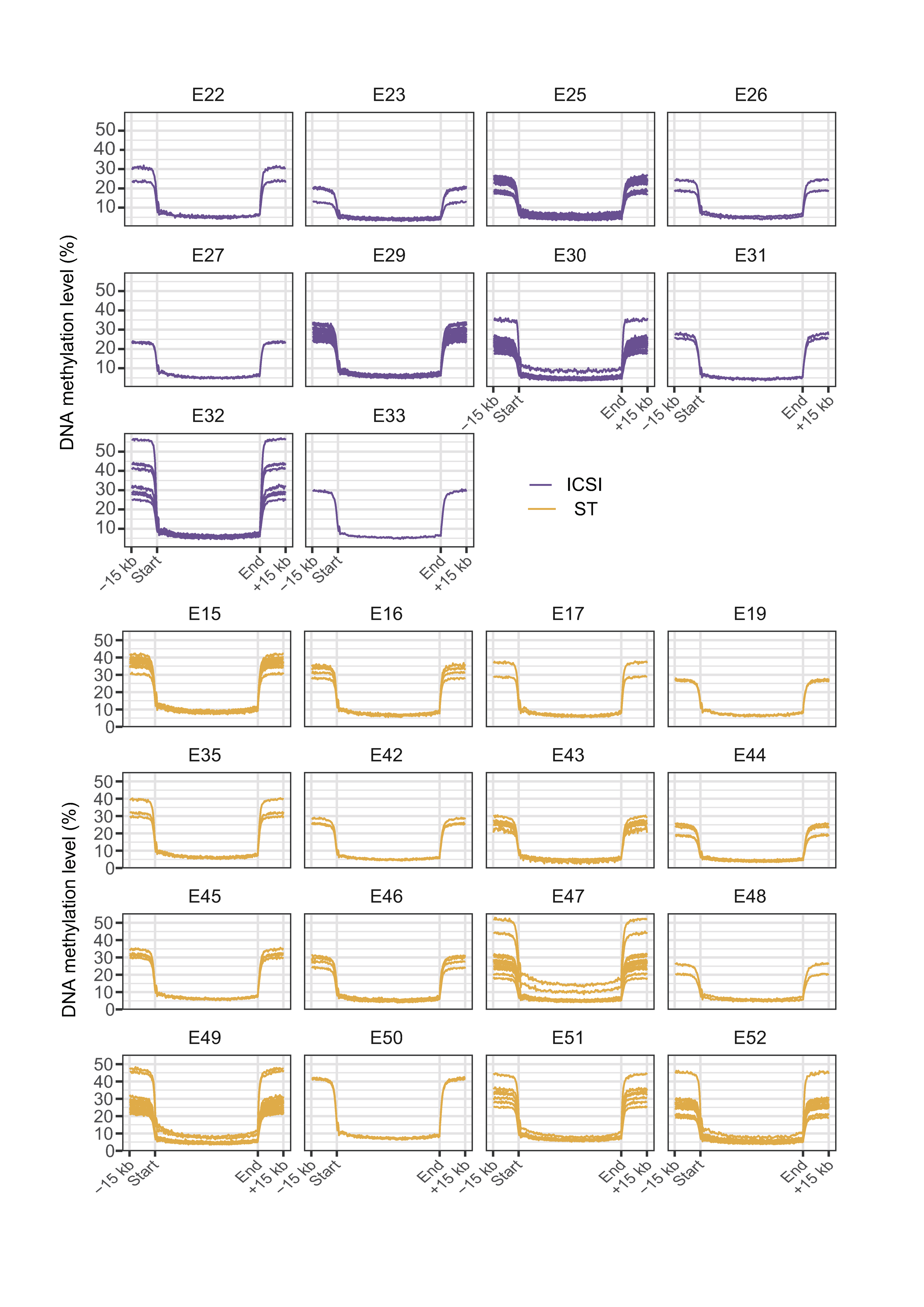

Supplement: S6 Fig — The numerical data are listed in S4 Data. CGI, CpG island; ICSI, intracytoplasmic sperm injection; ST, spindle transfer. (TIF) [file pbio.3001741.s006.tif]

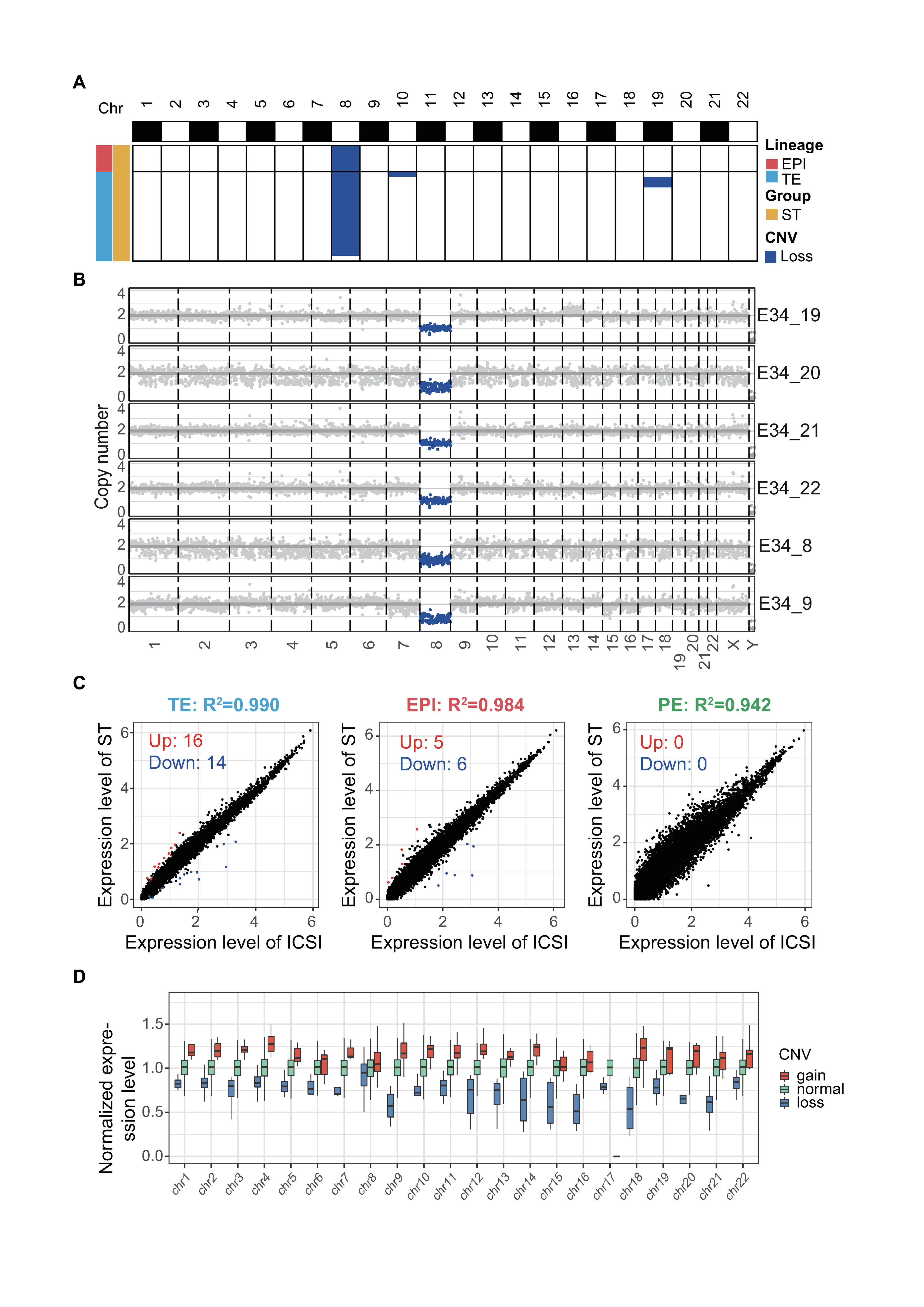

Supplement: S7 Fig — (A, B) Embryo E34 was selected to show that CNVs inferred from RNA expression levels (heatmap, A) and CNVs called from DNA methylome (dot plot, B) were consistent and can be used to validate each other. (C) Analysis of DEGs and linear regression analysis of euploid cells. (D) CNVs inferred from RNA expression levels were correlated across all of the chromosomes that comparing to the baseline (chromosomes with no CNVs, normal, green), chromosomes gaining copy numbers (red) have relative higher normalized expression levels while chromosomes losing copy numbers (blue) have relatively lower normalized expression levels. The numerical data are listed in S4 Data. CNV, copy number variation; DEG, differentially expressed gene; EPI, epiblast; ICSI, intracytoplasmic sperm injection; PE, primitive endoderm; ST, spindle transfer; TE, trophectoderm. (TIF) [file pbio.3001741.s007.tif]

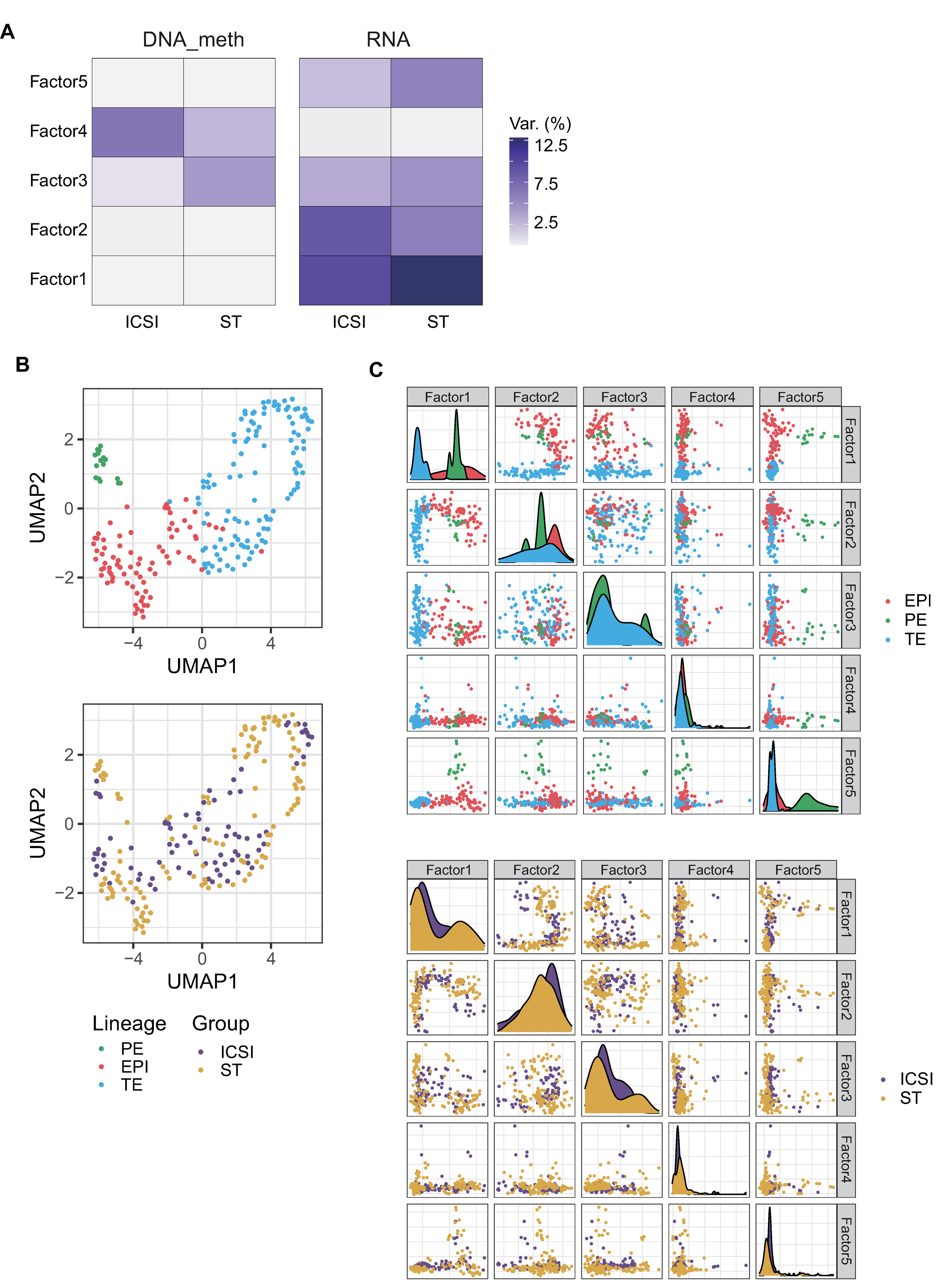

Supplement: S8 Fig — (A) Factors were calculated from integrating transcriptome (RNA, right panel) and DNA methylome (DNA_meth, left pane) by MOFA2 and ordered by their explaining variance (Var). (B) Cells were embedded into the low-dimensional space by UMAP and colored according to the cell lineage or group they belong to. (C) Combination of the top 5 factors explaining variance to visually show the separation of cells under the consideration of cell lineage or group. The numerical data are listed in S4 Data. EPI, epiblast; ICSI, intracytoplasmic sperm injection; PE, primitive endoderm; ST, spindle transfer; TE, trophectoderm; UMAP, uniform manifold approximation and projection. (TIF) [file pbio.3001741.s008.tif]
